# Full-thickness skin mesh graft vaginoplasty: a skin sparing technique

**DOI:** 10.1590/S1677-5538.IBJU.2016.0259

**Published:** 2017

**Authors:** Guilherme Lang Motta, Patric Machado Tavares, Gabriel Veber Moisés da Silva, Milton Berger, Brasil Silva, Tiago Elias Rosito

**Affiliations:** 1 Departamento de Urologia do Hospital das Clínicas de Porto Alegre, Universidade Federal do Rio Grande do Sul, RS, Brasil

## Abstract

**Introduction:**

The ideal vaginoplasty method should promote good cosmetic and functional results with low morbidity. We describe a new technique for congenital vaginal agenesis using a full-thickness perforated skin graft.

**Materials and Methods:**

We report an 18 year old patient with vaginal agenesis (Morris syndrome) that undergone a modified version of McIndoe vaginoplasty.

Patient is set in a low lithotomy position and lateral traction sutures are placed in labia and a 16Fr urethral catheter inserted. An inverted “V”-shaped incision is made in the mucosal plaque below the urethra. Blunt dissection in a cephalic posterior direction forms a space between the rectum and urethra. Special care is taken to avoid rectal tear during this maneuver. A full-thickness skin graft is removed from the lower abdomen measuring 12.0×6.0cm as an aesthetic abdominoplasty. The fat tissue is removed, remaining epidermis and dermis and the graft is perforated, allowing a great surface increase. After suturing over a mold, the graft is fixed in the created space. The donor site is closed with intradermal transversal suture.

**Results:**

From January 2009 to August 2015, seven patients diagnosed with vaginal agenesis underwent this technique. There were no major complications or need for blood transfusions. At the six-month follow-up, all patients reported satisfactory sexual intercourse. There were no significant complications at donor site or neovagina that needed surgical intervention.

**Conclusion:**

Vaginal reconstruction using the perforated graft is viable with excellent functional results. Applying this modification, we yielded the good results of a classic McIndoe technique with lower donor site morbidity.

## ARTICLE INFO

**Available at: http://www.intbrazjurol.com.br/video-section/20160259-motta_et_al/**

**Int Braz J Urol. 2017; 43 (Video #17): 1193-1193**

